# Homœopathy in Dental Practice

**Published:** 1893-06

**Authors:** Jules J. Sarrazie


ARTICLE III.

### HOMŒOPATHY IN DENTAL PRACTICE.

JULES J. SARRAZIE, D. D. S.

Read before the Louisiana State Dental Society, February, '93.

Five years ago I was first invited to experiment the effects of homœopathic treatment by a medical gentleman whose office was then across the street from mine. I was then a very strong disbeliever in the system, and carried my antagonism so far that whenever an opportunity presented itself, not only would I ridicule the treatment, but even did not spare the adept of it who chanced to have entered into a discussion with me on the subject. I had taken the trouble to figure out the fraction of a drop of tincture that entered into a ʒ of a third dilution, and the calculation had quite convinced me—thanks to my previous education in materia medica and therapeutics—that such infinitesimal quantities were absolutely incapable of producing any effect whatever on a living organism. This said for a third dilution, a low one, what then about a 12th, a 30th, a 200th, a 1000th, or higher still? The thing appeared irrational, foolish, stupid. Preconceived ideas are dangerous friends. I had been taught that drugs acted on our system by absorption into circulation, and that, in all instances, it required a certain quantity of them to produce an effect. Drug action had been studied, but nervous reaction against it, I was quite ignorant of, and even when compounded with the facts, long afterwards did my reason rebel against a therapeutical modus operandi, which it did not then comprehend, for lack of education in that direction, but which is, however, very simple. We are well aware that cathartics, for instance, will, as an after effect, that is after the action of the drug absorbed has thoroughly exhausted itself, produce constipation, and that the more frequently they are resorted to, and the more tenacious will constipation become when all treatment of this kind has been continued for enough time, so much so that people who accustom their digestive apparatus to regulating pills, laxatives and the like, become, in time, slaves to them, until they cannot live comfortably without them. Also persons who contract the habit of using morphine to induce sleep, will, after a time, become victims to the habit, because natural sleep will fail them, and rest must be procured by drug effect. Again, the tobacco, coffee or alcohol consumer feels his system so depressed at every attempt to discontinue these several stimulants, that he resorts to habitual and constant indulgence to obtain the required drug effect to prevent the nervous reaction which must set in when the stimulant is no longer indulged in. It is upon these facts that homœopathic treatment rests; on the nervous reaction brought about by the administration of curative agents, in the aid thus offered our vital force to react against disease and conquer it. We all know that vital force alone is the power which restores health; a stimulant to it, a push. I may say, to cause nervous reaction in the proper direction, is the result aimed at; the why and wherefore of the formula: "Similia Simibus." If we administer a medicine, the drug effects of which would produce symptoms similar to those of the patient, frequently repeated in such infinitesimal doses as cannot possibly produce an aggravation of the ailments we are fighting, we must necessarily awaken the dormant vital force to react against the symptomatology of the curative agent and the disease at the same time, and the battle is won, provided sufficient oil remains in the lamp and the proper agent has been selected to cover the symptoms of the individual case and all the peculiarities of each particular patient. It is in this selection that comes the great difficulty; it is here that errors cause failures, particularly so when it is almost impossible to obtain, as specialists like we, all the information possible regarding the general health of the patient, and are compelled to prescribe for conditions which we see, when affinity elsewhere in the organism may be greater for the remedy we select than even that acute condition which we are called upon to alleviate, and even then I do claim better, surer and more lasting results can be obtained by this means than by any other. For instance, I will say that I have not yet observed alveolar abscess to recur where I had treated it homœopathically; that the per centage of failure in acute or chronic cases is so small, that, considering in the first place the difficulties before mentioned, under which a dental practitioner must prescribe, and secondly, the possibility of error in the selection of the desired curative agent, that I do not hesitate to ascribe those two causes the rare failure themselves, since the lack of reactive vital energy is hardly ever found in such cases, our patients not ordinarily being debilitated invalids. Of course, some few, very few indeed, acute cases resist treatment, and to give relief we must take refuge in the old time method of extraction, but where such is the case, we can safely assert that some local mechanical cause, such as the formation of gases in curved, inaccessible root, or still yet some other cause of irritation is at work against us. This much said about acute cases, still better and more surprising results can be obtained in old chronic ones, where all previous efforts have entirely failed. Unfortunately, gentlemen, the selection of the remedy best indicated from individual case is often no easy matter, and although I will soon depict a few pathological conditions, and indicate their treatment, this may vary somewhat, according to circumstances.

In order not to confuse my subject I will consider only one condition of the organs we are accustomed to deal with, periostitis and alveolar abscess acute and chronic, leaving entirely aside all other diseases of the oral cavity, and even here some little study is necessary before any amount of accuracy in prescribing can be obtained. Right here already a little list of remedies suggest themselves, ready to puzzle the beginner, and from which we must select with great care and precision considering at times even the cause of the trouble if ascertainable and the constitution and disposition of the patient. Aconitum, mercurius dulcamara, belladonna, arnica chammomilla, calcarea carbonica fluorica or phosphorica, acidum fluoricum, sulphur, hepar sulphur and silicea would be a few of those most likely to be called into active service in fighting different forms of alveolar abscess and its after results when the patient has not been seen in time. Taking periostitis as a starting point a few of the above may be selected from. Mercurius, if soreness upon pressure with or without spongy inflammation of the gums, is manifested, particularly if symptoms of cold in the head and in the throat concur, and we ascertain that the patient has taken cold. This, provided however, that the patient is not suffering from the drug effects of mercurial preparations, in which case hepar sulphur being the antidote of mercurius, should be given. Aconitum may be used with advantage in periostitis if there is much inflammation, some fever, cold has been taken, and relief is procured by cold applications. If the trouble can be ascribed to the effects of having been wet, and there is at the same time some pulpitis, dulcamara will be found valuable. Belladonna will apply in periostitis where inflammation is marked, and headache in the front portion of the cranium is manifested. Arnica is clearly indicated if the cause of the trouble is previous concussion or mechanical pressure in any manner chammomilla, with soreness and pulsating pain, has the sensatory symptom of elongation of the tooth very marked; warm applications relieve. Any two of the above mentioned remedies may be alterned if so indicated. In this incipient form of periostitis a medium dilution, say the 30th, is preferable, particularly if the patient uses neither coffee nor tobacco in any quantity, and if total abstention from these nervous stimulants can be insured, so much the better, otherwise, lower dilutions, the 6th, for instance, can be displayed. This much said about incipiency, let us now pass to a better defined stage. We have considered periostitis singly, whether due to mechanical irritation, cold or incipient abscess. In the latter case, we may, instead of resolution, have a tendency towards pus formation, characterized by the symptoms of heat, painful pulsation, swelling, redness, soreness and elongation still better marked than in the conditions above described. The remedy is now hepar sulphur, unless the patient is syphilitic or shows syphilitic heredity, in which case mercurius virus should be given the preference, except in case the system is already saturated with mercury. Mercurius if chosen for the reasons above stated will usually lend towards resolution. Hepar sulphur may do likewise, or may hasten suppuration in a surprising degree. In neither case will there be much suffering. Mercurius and hepar sulphur being antidotes should not be alternated, of course. Low dilutions of hepar sulph, the 6th for instance, will tend toward suppuration; medium, like the 30th, will on the other hand tend toward resolution. In making choice of the dilution, should be well observed, as previously stated, the patient's mode of life and habits. The next stage of alveolar abscess is that in which the formation of pus however slight has already started in. This is characterized by the peculiar feeling of the parts well-known to all dental practitioners, less pulsation, swelling, bloated appearance. Silicea should be exhibited and prescribed at a high potency in preference if the habits of the patient allow it. This concludes the treatment of acute conditions, taken at any stage of the trouble, and brought to its termination, and I would here state that cases so treated will not need to be followed farther into those chronic phases which will now be considered. Of course, mechanical means, such as affording free exit to the gases of decomposed pulp matter, and washing root canals with injections of hydrogen peroxide, should be at all times resorted to. I always prefer hydrogen peroxide to other disinfectants, because of its detergent qualities combined with medicinal inertness which does not imperil the action of the remedies employed. For the sake of clearness I will now describe some of the chronic conditions which may result from alveolar abscess, under the headings of those remedies which may there apyly:

Calcarea, Carbonica or Phosphorica.—Looseness of the tooth caused by absorption of the alveolus.

Calcarea Fluorica.—The same conditions as above; the root being somewhat of a foreign substance, owing either to lack of vitality of its pericementum or to exposure by absorption at its apex of some of the enclosed filling material.

Acidum Fluoricum.—Lack of life and adhesion of the roots caused by the presence of foreign matter at the apex or partial death of the lining membrane; fistula, discharge of pus therefrom and necrosis.

Silicea.—Looseness of the tooth, fistula, discharge of pus.

Sulphur.—Well adapted to most cases in the beginning of treatment in chronic cases to put the system in a better condition to react. For this purpose, should be prescribed at a high potency in preference, about the 200th.

This, gentlemen, is the homœopathic treatment which will usually apply to ordinary cases of alveolar abscess in its different stages. In order not to make this article too long and complicated, I have purposely avoided any allusion to other diseases of the oral cavity and adjoining parts; pulpitis, gingivitis, recession, resorption, porrhœa, facial neuralgia and the like. For all such cases, as well as for those pathological conditions here depicted, I will at any time be happy to give snggestions and information in regard to the remedy best adapted, and thereby facilitate the trial of a system of treatment, which I am convinced, would if more extensively used prove a boon both to ourselves and patients.—*Southern Dental Journal.*

